# The combined effect of triglyceride–glucose index and high‐sensitivity C‐reactive protein on cardiovascular outcomes in patients with chronic coronary syndrome: A multicenter cohort study

**DOI:** 10.1111/1753-0407.13589

**Published:** 2024-08-13

**Authors:** Qinxue Li, Ying Song, Zheng Zhang, Jingjing Xu, Zhenyu Liu, Xiaofang Tang, Xiaozeng Wang, Yan Chen, Yongzhen Zhang, Pei Zhu, Xiaogang Guo, Lin Jiang, Zhifang Wang, Ru Liu, Qingsheng Wang, Yi Yao, Yingqing Feng, Yaling Han, Jinqing Yuan

**Affiliations:** ^1^ National Clinical Research Center for Cardiovascular Diseases, State Key Laboratory of Cardiovascular Disease, Fuwai Hospital, National Center for Cardiovascular Diseases Chinese Academy of Medical Sciences and Peking Union Medical College Beijing China; ^2^ Department of Cardiology The First Hospital of Lanzhou University Lanzhou China; ^3^ Department of Cardiology, Peking Union Medical College Hospital Chinese Academy of Medical Sciences and Peking Union Medical College Beijing China; ^4^ Department of Cardiology General Hospital of Northern Theater Command Shenyang China; ^5^ Department of Cardiology Peking University Third Hospital Beijing China; ^6^ Department of Cardiology, The First Affiliated Hospital Zhejiang University School of Medicine Zhejiang China; ^7^ Department of Cardiology Xinxiang Central Hospital Xinxiang China; ^8^ Department of Cardiology The First Hospital of Qinhuangdao Qinhuangdao China; ^9^ Department of Cardiology Guangdong Provincial People's Hospital Guangdong China

**Keywords:** chronic coronary syndrome (CCS), high‐sensitivity C‐reactive protein (hsCRP), major adverse cardiovascular events (MACE), triglyceride–glucose (TyG) index

## Abstract

**Background:**

The triglyceride–glucose (TyG) index and high‐sensitivity C‐reactive protein (hsCRP) are the commonly used biomarkers for insulin resistance and systemic inflammation, respectively. We aimed to investigate the combined association of TyG and hsCRP with the major adverse cardiovascular events (MACE) in patients with chronic coronary syndrome (CCS).

**Methods:**

A total of 9421 patients with CCS were included in this study. The primary endpoint was defined as a composite of MACE covering all‐cause death, nonfatal myocardial infarction, and revascularization.

**Results:**

During the 2‐year follow‐up period, 660 (7.0%) cases of MACE were recorded. Participants were divided equally into three groups according to TyG levels. Compared with the TyG T1 group, the risk of MACE was significantly higher in the TyG T3 group. It is noteworthy that among patients in the highest tertile of TyG, hsCRP >3 mg/L was significantly associated with an increased risk of MACE, whereas the results were not significant in the medium to low TyG groups. When patients were divided into six groups according to hsCRP and TyG, the Cox regression analysis showed that patients in the TyG T3 and hsCRP >3 mg/L group had a significantly higher risk of MACE than those in the TyG T1 and hsCRP ≤3 mg/L group. However, no significant interaction was found between TyG and hsCRP on the risk of MACE.

**Conclusion:**

Our study suggests that the concurrent assessment of TyG and hsCRP may be valuable in identifying high‐risk populations and guiding management strategies among CCS patients.

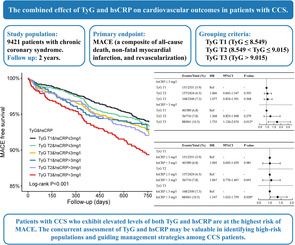

## Graphical abstract

Highlights

- A large‐scale study investigating the joint prognostic effect of TyG and hsCRP in patients with CCS.
- Patients with CCS who exhibit elevated levels of both TyG and hsCRP are at the highest risk of MACE.
- Simultaneous assessment of TyG and hsCRP may be valuable in identifying the high‐risk populations and guiding management strategies among patients with CCS.

## 1 INTRODUCTION

Coronary artery disease (CAD) is one of the most important diseases that threaten the health of humans. Despite the progressive advancement of pharmacologic and revascularization therapies, patients with CAD are still at a relatively high risk for adverse cardiovascular events.^1^ Therefore, it is essential to clarify the residual cardiovascular risk. CAD is currently divided into two categories, namely acute coronary syndrome (ACS) and chronic coronary syndrome (CCS), in which CCS is the main population.^2^

Insulin resistance, defined as a reduction in insulin sensitivity, is one of the major risk factors for CAD.^3^, ^4^ Using the hyperinsulinemic–euglycemic clamp to measure insulin resistance is invasive and difficult to obtain. Triglyceride–glucose (TyG) index calculated from fasting triglyceride (TG) and fasting blood glucose (FBG) is currently a proven noninvasive alternative indicator of insulin resistance.^5^, ^6^ A growing amount of evidence has shown that the TyG index independently predicts the occurrence of CAD^7^, ^8^ and is independently associated with poor prognosis in patients with CAD.^9^, ^10^, ^11^ However, most of the current studies in patients with CAD have focused on the population with ACS and undergoing percutaneous coronary intervention (PCI), and there are fewer studies on TyG and the occurrence of adverse cardiovascular events in patients with CCS.

Inflammation is another important influential factor in the development of CAD.^12^ High‐sensitivity C‐reactive protein (hsCRP), a commonly used biomarker reflecting systemic inflammation, is an important and independent predictor of CAD pathogenesis and prognosis.^13^, ^14^, ^15^, ^16^ There is a tight interconnection between insulin resistance and inflammation.^17^, ^18^ A recent study of middle‐aged and elderly Chinese individuals showed that participants with high hsCRP and TyG had a higher risk of cardiovascular disease than those with low hsCRP and TyG.^19^ However, fewer studies have targeted the correlation between TyG combined with hsCRP and the prognosis of patients with confirmed CAD.

Therefore, in this study, we aimed to investigate the association of the TyG index with the risk of the major adverse cardiovascular events (MACE) in patients with CCS and to further investigate the combined association of TyG and hsCRP with the MACE.

## 2 METHODS

### 2.1 Study population

**FIGURE 1:**
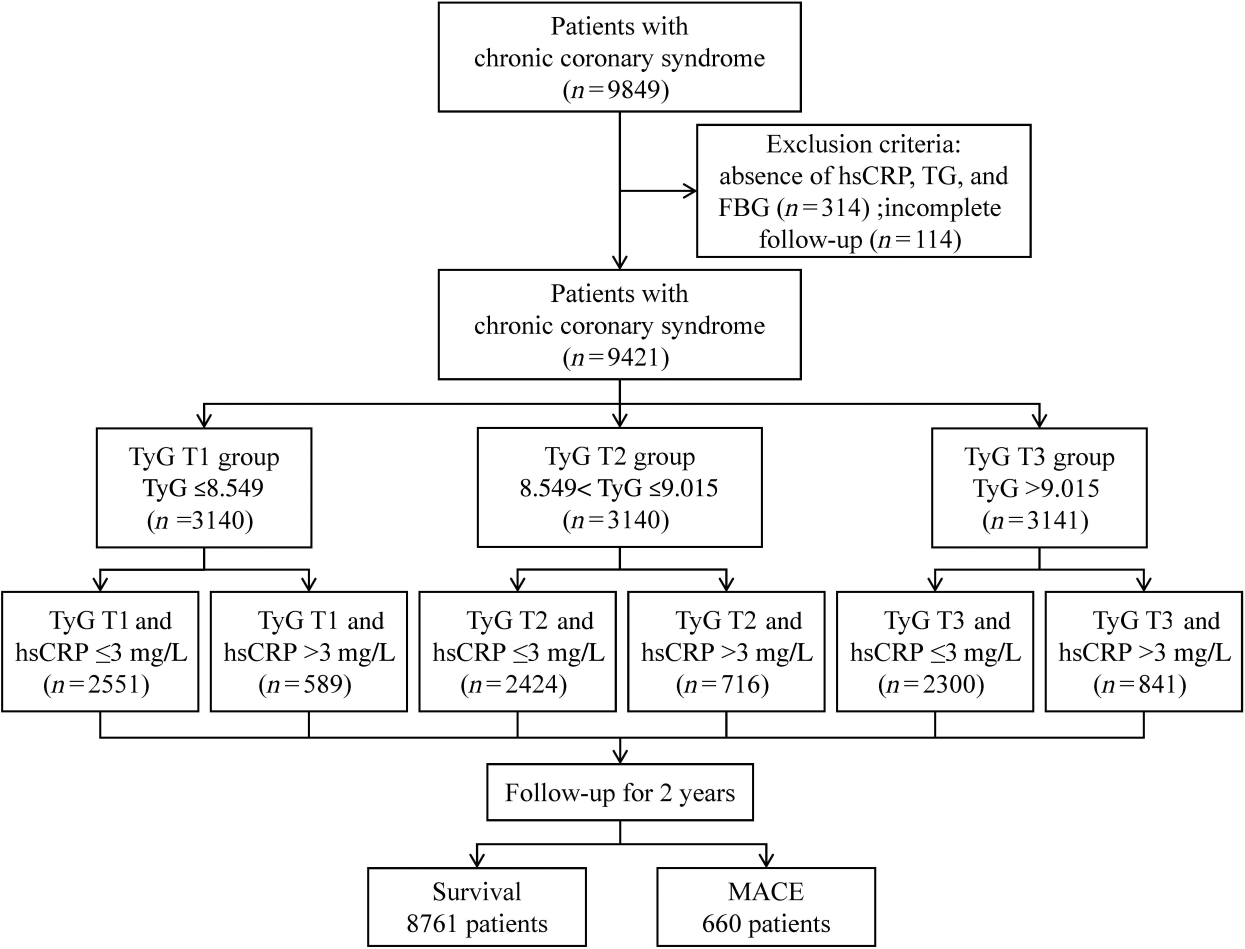
Flowchart of the study. FBG, fasting blood glucose; hsCRP, high‐sensitivity C‐reactive protein; MACE, major adverse cardiovascular events; TG, triglyceride; TyG, triglyceride–glucose.

The present study used data from the PRospective Observational Multi‐center cohort for ISchemic and hEmorrhage risk in coronary artery disease patients (PROMISE). The PROMISE study was conducted on 18 701 patients with confirmed CAD who were hospitalized at nine medical centers in China from January 2015 to May 2019. Among them, 9849 patients met the inclusion criteria of the present study: diagnosed with chronic coronary syndrome^2^; older than or equal to 18 years of age; and voluntarily signed an informed consent form. Exclusion criteria included the absence of hsCRP, TG, and FBG (*n* = 314) and incomplete follow‐up (*n* = 114). Ultimately, a total of 9421 patients with chronic coronary syndrome were included in this study. At first, patients were divided equally into three groups according to their TyG levels: T1 group (TyG ≤8.549), T2 group (8.549< TyG ≤9.015), and T3 group (TyG >9.015). To further investigate the correlation between TyG combined with hsCRP and the poor prognosis, patients were divided into six groups according to the levels of hsCRP and TyG: TyG T1 and hsCRP ≤3 mg/L group, TyG T2 and hsCRP ≤3 mg/L group, TyG T3 and hsCRP≤3 mg/L group, TyG T1 and hsCRP >3 mg/L group, TyG T2 and hsCRP >3 mg/L group, and TyG T3 and hsCRP >3 mg/L group. The research flow chart is detailed in Figure 1. This study complied with the principles of the Declaration of Helsinki. The ethics committee/institutional review board at each study site approved the study protocol. All participants provided written informed consent before enrollment.

### 2.2 Data collection and definitions

Patients' peripheral venous blood samples were collected in the morning after an overnight fast following admission to the hospital for the measurement of biochemical parameters. During hospitalization, the experienced cardiology team selected either PCI or coronary artery bypass grafting (CABG) for hemodynamic reconstruction. Patients' information was collected from the electronic medical record, which included demographic information, clinical characteristics, medications, ultrasound cardiogram, and coronary angiography details. All statistics were verified and managed by independent statisticians.

Hypertension was defined as a self‐reported history of hypertension, use of antihypertensive medications, systolic blood pressure ≥140 mmHg, or diastolic blood pressure ≥90 mmHg. Diabetes was diagnosed by FBG ≥7.0 mmol/L, hemoglobin A1c levels ≥6.5%, or 2‐h blood glucose of oral glucose tolerance test ≥11.1 mmol/L. Left main artery disease was defined as ≥50% stenosis of the left main coronary artery, and three‐vessel disease was defined as ≥50% stenosis in three major coronary branches (vessel diameter ≥2 mm). The TyG index was calculated as follows: ln [fasting TGs (mg/dL) × FBG (mg/dL)/2].^5^

### 2.3 Follow‐up and endpoint event

All enrolled patients were followed up at 1 year and 2 years after discharge through outpatient visits, phone calls, text messages, and letters. To ensure the quality of follow‐up interviews, clinical research coordinators received professional training, recorded follow‐up calls, and used a unified online follow‐up system. All endpoint events were checked by two experienced and independent clinicians, and disagreements were resolved by consensus. The primary endpoint was defined as a composite of the MACE covering all‐cause death, nonfatal myocardial infarction, and revascularization. The secondary outcome was each component of MACE. All‐cause mortality was defined as death for any cause. Myocardial infarction was diagnosed in accordance with the contemporaneous Universal Definition of Myocardial Infarction.^20^, ^21^ Cardiac death was defined as death due to a proximate cardiovascular cause or any death without an unequivocal noncardiovascular cause.^22^, ^23^ Revascularization included any ischemia‐driven repeat PCI or bypass surgery.

### 2.4 Statistical analysis

Continuous variables were expressed as mean ± standard deviation and comparisons were made between multiple groups using the analysis of variance test or Kruskal–Wallis *H* test. Categorical variables were expressed as counts (percentages) and compared between groups using the chi‐square test or Fisher's exact probability method. The Kaplan–Meier method was used to draw survival analysis curves, and log‐rank analysis was performed to compare the differences in the incidence of follow‐up endpoint events between groups. The hazard ratio (HR) and 95% confidence interval (CI) of risk factors associated with endpoint events were analyzed using univariate and multivariate Cox regression models. The confounding factors adjusted in the multivariate Cox regression model included clinically relevant variables or those that showed statistical significance in the univariate Cox regression analysis. Restricted cubic splines (RCS) were used to visualize the relationship between TyG, hsCRP, and poor prognosis. In addition, Cox regression was employed to assess the multiplicative interaction between hsCRP and TyG. The approach proposed by Henrik Källberg et al.^24^ was utilized to evaluate the additive interaction, comprising three metrics: RERI (relative excess risk due to interaction), AP (proportion attributable to interaction), and SI (synergy index). Further sensitivity analysis was performed to explore the consistency of the results by regrouping according to the median level and 2 mg/L of hsCRP. Statistical analyses were performed using IBM SPSS software (version 26.0) and R Programming Language (version 4.2.2), and two‐tailed *p* value <0.05 was regarded as statistically significant.

## 3 RESULTS

### 3.1 Baseline characteristics

A total of 9421 patients (61.05 ± 9.71 years, 73.1% male) who met the criteria and completed the follow‐up were finally enrolled in this study and divided into three groups according to their TyG levels, and the baseline characteristics of each group were shown in Table 1. The patients with higher TyG levels appear to be younger, less male, and with higher body mass index (BMI). The patients with higher TyG levels had higher proportions of current smoking, hypertension, hyperlipidemia, diabetes, and previous myocardial infarction. Patients with high TyG had higher laboratory indexes, including hsCRP, TGs, TC, low‐density lipoprotein cholesterol (LDL‐C), creatine, uric acid, FBG, glycosylated hemoglobin A1c (HbA1c), and albumin, and they had lower high‐density lipoprotein cholesterol levels. Participants with higher TyG had a higher incidence of triple‐vessel disease and higher synergy between PCI with taxus and cardiac surgery (SYNTAX) scores compared with participants with lower TyG.

**TABLE 1:** Baseline characteristics of different TyG groups.

Variables	TyG T1 (*n* = 3140)	TyG T2 (*n* = 3140)	TyG T3 (*n* = 3141)	*p* value
TyG	8.23 ± 0.25	8.78 ± 0.13	9.47 ± 0.42	<0.001*
Demographic characteristics				
Age, years	61.96 ± 9.99	61.19 ± 9.50	60.00 ± 9.53	<0.001*
BMI, kg/m^2^	25.09 ± 3.32	26.15 ± 3.27	26.72 ± 3.06	<0.001*
Male, %	2410 (76.8)	2276 (72.5)	2201 (70.1)	<0.001*
Coexisting conditions, %				
Current smoking	425 (13.5)	529 (16.8)	646 (20.6)	<0.001*
Hypertension	1948 (62.0)	2172 (69.2)	2258 (71.9)	<0.001*
Hyperlipidemia	2695 (85.8)	2767 (88.1)	2797 (89.0)	<0.001*
DM	1020 (32.5)	1371 (43.7)	2059 (65.6)	<0.001*
Cerebrovascular diseases	507 (16.1)	499 (15.9)	524 (16.7)	0.686
COPD	56 (1.8)	47 (1.5)	36 (1.1)	0.111
Anemia	36 (1.1)	24 (0.8)	29 (0.9)	0.290
Previous myocardial infarction	581 (18.5)	606 (19.3)	661 (21.0)	0.035*
Previous PCI	911 (29.0)	914 (29.1)	934 (29.7)	0.791
Previous CABG	83 (2.6)	85 (2.7)	94 (3.0)	0.669
Peripheral vascular diseases	233 (7.4)	215 (6.8)	209 (6.7)	0.463
Laboratory tests				
hsCRP, mg/L	2.24 ± 3.67	2.46 ± 3.13	2.75 ± 3.46	<0.001*
TG, mg/mL	83.40 ± 19.59	128.83 ± 26.85	223.67 ± 123.29	<0.001*
TC, mmol/L	3.65 ± 0.94	4.01 ± 0.97	4.41 ± 1.12	<0.001*
HDL‐C, mmol/L	1.24 ± 0.33	1.14 ± 0.29	1.05 ± 0.26	<0.001*
LDL‐C, mmol/L	2.11 ± 0.80	2.41 ± 0.85	2.58 ± 0.91	<0.001*
Creatine, μmol/L	80.31 ± 26.37	80.67 ± 16.69	82.97 ± 21.84	<0.001*
Uric acid, μmol/L	331.23 ± 81.76	345.95 ± 85.61	356.07 ± 94.92	<0.001*
FBG, mg/mL	93.66 ± 16.52	105.22 ± 23.17	135.12 ± 50.90	<0.001*
HbA1c, %	6.02 ± 1.28	6.29 ± 0.94	7.06 ± 1.55	<0.001*
Albumin, g/L	43.70 ± 4.52	44.95 ± 4.34	45.33 ± 4.33	<0.001*
Angiographic details, %				
LM	343 (10.9)	362 (11.5)	359 (11.4)	0.719
TVD	1160 (36.9)	1255 (40.0)	1420 (45.2)	<0.001*
Bridge vascular lesions	46 (1.5)	54 (1.7)	65 (2.1)	0.186
SYNTAX score	11.99 ± 8.62	12.16 ± 8.61	12.97 ± 8.86	<0.001*
LVEF, %	61.49 ± 7.14	61.80 ± 6.84	61.69 ± 7.00	0.482
IABP use, %	15 (0.5)	10 (0.3)	10 (0.3)	0.488

Abbreviations: BMI, body mass index; CABG, coronary artery bypass grafting; COPD, chronic obstructive pulmonary disease; DM, diabetes mellitus; FBG, fasting blood glucose; HbA1c, glycosylated hemoglobin A1c; HDL‐C, high‐density lipoprotein cholesterol; hsCRP, high‐sensitivity C‐reactive protein; IABP, intra‐aortic balloon pump; LDL‐C, low‐density lipoprotein cholesterol; LM, left main; LVEF, left ventricular ejection fraction; PCI, percutaneous coronary intervention; SYNTAX, synergy between PCI with taxus and cardiac surgery; TC, total cholesterol; TG, triglyceride; TVD, three‐vessel disease; TyG, triglyceride–glucose.

* *p* value <0.05.

### 3.2 TyG levels and clinical endpoints

**FIGURE 2:**
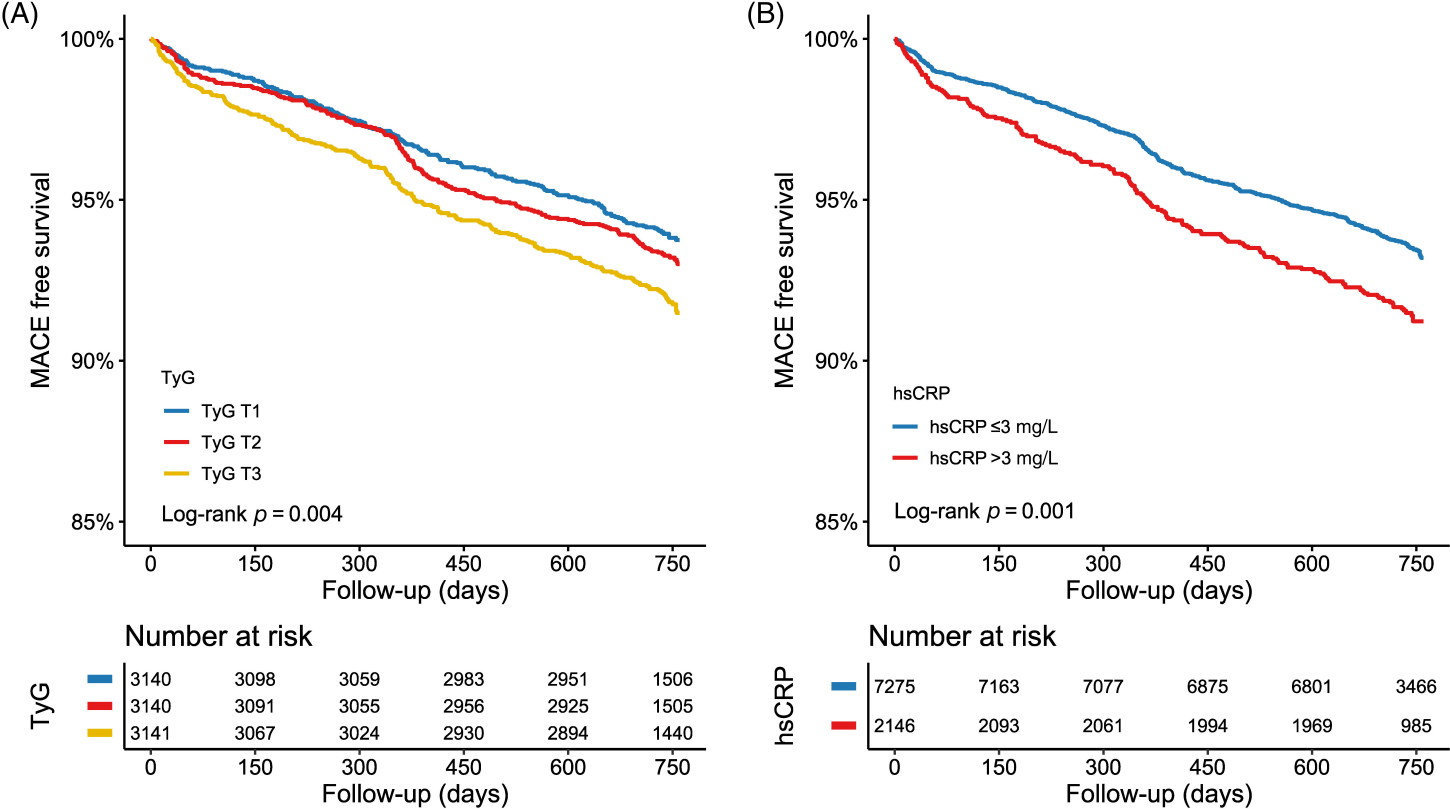
Kaplan–Meier analysis for major adverse cardiovascular events (MACE) according to triglyceride–glucose (TyG) and high‐sensitivity C‐reactive protein (hsCRP).

**FIGURE 3:**
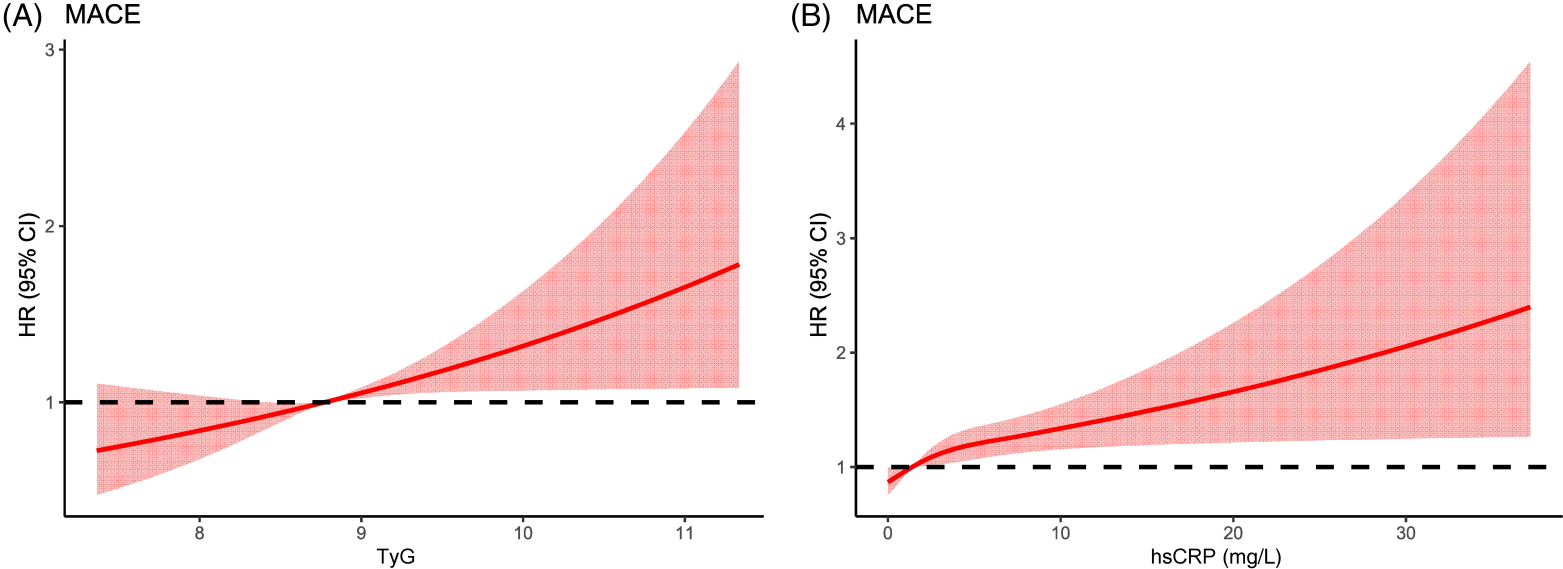
Restricted cubic spline curves of triglyceride–glucose (TyG) and high‐sensitivity C‐reactive protein (hsCRP) for major adverse cardiovascular events (MACE). CI, confidence interval; HR, hazard ratio.

During the 2‐year follow‐up period (interquartile range: 2.0–2.1 years), 660 (7.0%) cases of MACE were recorded. The incidence of endpoint events differed among participants in different TyG groups, with a significantly higher incidence of MACE in the T3 group (6.1%, 6.8%, and 8.2% in the T1, T2, and T3 groups, respectively). Kaplan–Meier curves showed similar results, with a significantly higher cumulative risk of composite MACE in the T3 group than the other two groups (Log‐rank *p* < 0.05) (Figure 2A). Three Cox regression models were developed to assess the correlation between TyG and the risk of MACE, as shown in Table 2. Model 1: univariate model. Model 2: adjusted for sex, age, and diabetes mellitus (DM). Model 3: adjusted for sex, age, BMI, hypertension, cerebrovascular disease, peripheral vascular diseases, anemia, DM, previous myocardial infarction, previous PCI, previous CABG, LDL‐C, creatine, albumin, HbA1c, left ventricular ejection fraction, left main or three‐vessel disease, and SYNTAX score. The risk of MACE was significantly higher in the T3 group than in the T1 group in all three models (HR: 1.359, 95% CI: 1.127–1.639, *p* = 0.001; adjusted HR: 1.341, 95% CI: 1.102–1.632, *p* = 0.003; adjusted HR: 1.283, 95% CI: 1.037–1.587, *p* = 0.022). The same results can be observed by fitting the model using RCS and visualizing the relationship between TyG and poor prognosis (Figure 3).

**TABLE 2:** Univariate and multivariate cox model analysis for MACE across different TyG groups and hsCRP groups.

	Model 1			Model 2			Model 3		
	HR	95% CI	*p* value	HR	95% CI	*p* value	HR	95% CI	*p* value
TyG index									
Per unit increase	1.253	1.107–1.419	<0.001*	1.244	1.091–1.418	0.001*	1.214	1.050–1.404	0.009*
TyG T1	Ref	‐	‐	Ref	‐	‐	Ref	‐	‐
TyG T2	1.120	0.921–1.361	0.256	1.125	0.924–1.370	0.239	1.138	0.928–1.396	0.213
TyG T3	1.359	1.127–1.639	0.001*	1.341	1.102–1.632	0.003*	1.283	1.037–1.587	0.022*
hsCRP									
Per unit increase	1.029	1.015–1.045	<0.001*	1.030	1.015‐1.045	<0.001*	1.020	1.002–1.039	0.027*
hsCRP ≤3 mg/L	Ref	‐	‐	Ref	‐	‐	Ref	‐	‐
hsCRP >3 mg/L	1.329	1.121–1.575	0.001*	1.316	1.110–1.561	0.002*	1.179	0.987–1.409	0.069

*Note*: Model 1: univariate model. Model 2: adjusted for sex, age, and DM. Model 3: adjusted for sex, age, BMI, hypertension, cerebrovascular disease, peripheral vascular diseases, anemia, DM, previous myocardial infarction, previous PCI, previous CABG, LDL‐C, creatine, albumin, HbA1c, LVEF, LM/TVD, and SYNTAX score.

Abbreviations: BMI, body mass index; CABG, coronary artery bypass grafting; CI, confidence interval; DM, diabetes mellitus; HbA1c, glycosylated hemoglobin A1c; HR, hazard ratio; hsCRP, high‐sensitivity C‐reactive protein; LDL‐C, low‐density lipoprotein cholesterol; LM/TVD, left main or three‐vessel disease; LVEF, left ventricular ejection fraction; MACE, major adverse cardiovascular events; PCI, percutaneous coronary intervention; SYNTAX, synergy between PCI with taxus and cardiac surgery; TyG, triglyceride–glucose.

* *p* value <0.05.

### 3.3 TyG combined with hsCRP and clinical endpoints

**FIGURE 4:**
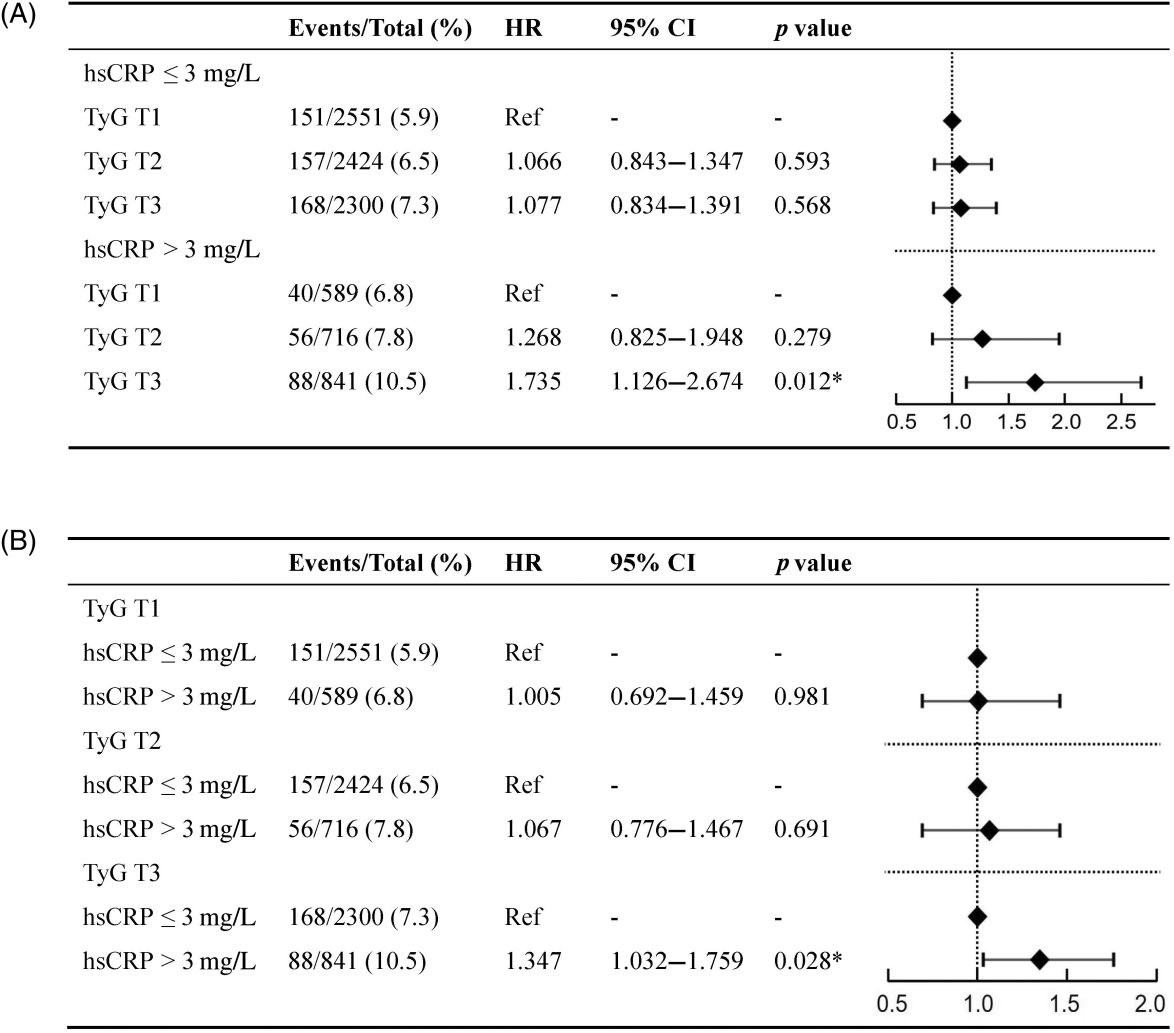
(A) The risk of major adverse cardiovascular events (MACE) across different triglyceride–glucose (TyG) levels stratified by high‐sensitivity C‐reactive protein (hsCRP). (B) The risk of MACE across different hsCRP levels stratified by TyG. CI, confidence interval; HR, hazard ratio. *p value <0.05.

Participants were divided into two groups based on the hsCRP level of 3 mg/L. Kaplan–Meier analysis showed that patients in the hsCRP >3 mg/L group had a significantly higher cumulative risk of MACE (Log‐rank *p* < 0.05) (Figure 2B). The correlation between TyG and MACE was assessed in different hsCRP groups. The results of the multivariate Cox regression model showed that the risk of MACE was significantly higher in the TyG T3 group with a higher level of hsCRP (adjusted HR: 1.735, 95% CI: 1.126–2.674, *p* = 0.012). However, the association between TyG and MACE was attenuated with lower hsCRP levels (Figure 4A). Furthermore, the correlation between hsCRP and MACE was evaluated in different TyG groups. The results revealed a significant association between hsCRP >3 mg/L and an increased risk of MACE among patients with the highest TyG tertile (adjusted HR: 1.347, 95% CI: 1.032–1.759; *p* = 0.028) (Figure 4B).

**FIGURE 5:**
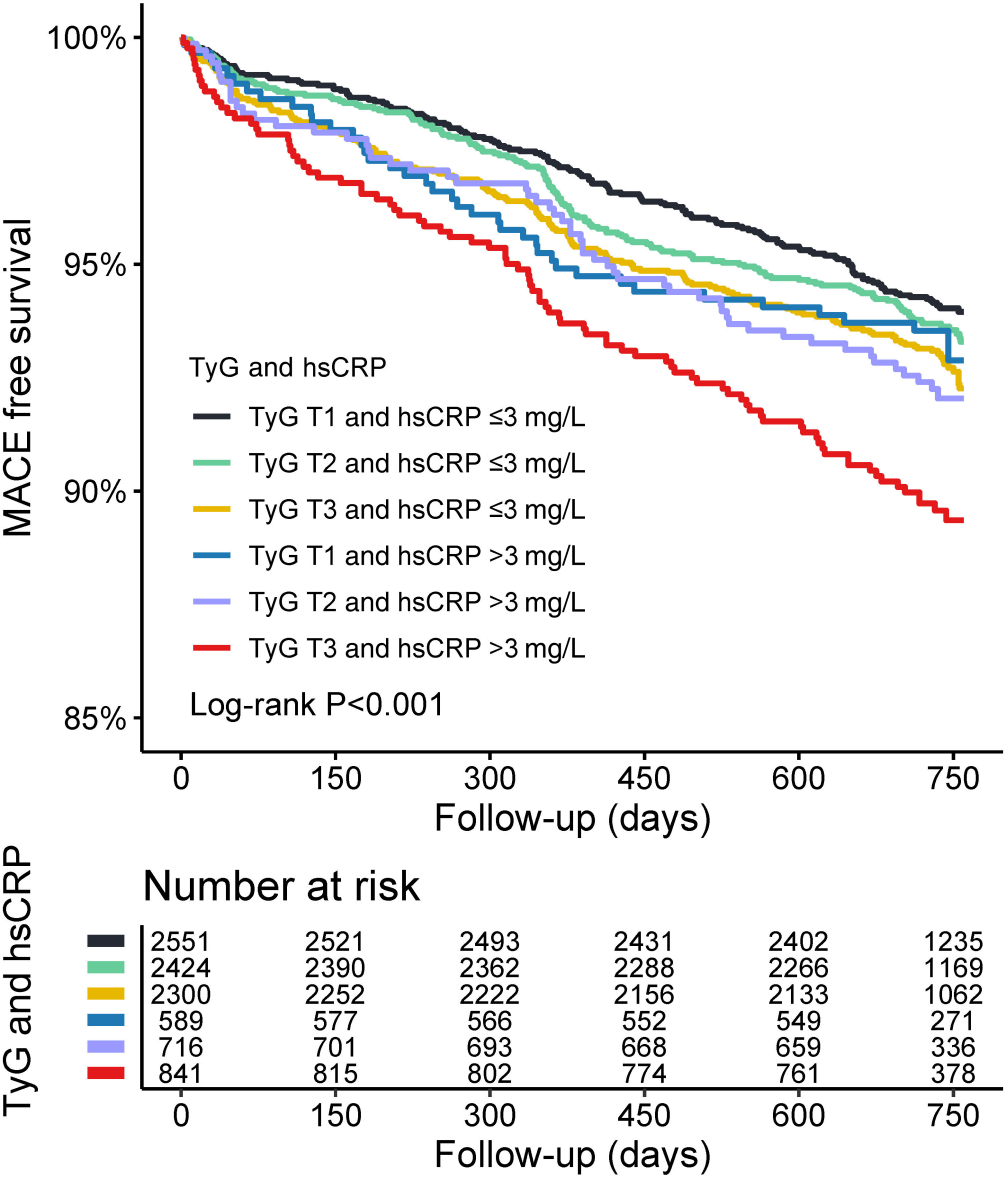
Kaplan–Meier analysis for major adverse cardiovascular events (MACE) according to combined groups stratified by triglyceride–glucose (TyG) and high‐sensitivity C‐reactive protein (hsCRP).

Patients were divided into six groups according to the levels of hsCRP and TyG for further investigation. Kaplan–Meier curves showed the highest cumulative risk of MACE in the TyG T3 and hsCRP >3 mg/L group (Log‐rank *p* < 0.05) (Figure 5). Multivariate Cox regression analysis showed that after adjusting for stepwise selection and clinically important variables, patients in the TyG T3 and hsCRP >3 mg/L group had a significantly higher risk of MACE (adjusted HR: 1.577, 95% CI: 1.180–2.108, *p* = 0.002) (Table 3). The Cox regression analysis of secondary endpoints showed that all‐cause mortality and nonfatal myocardial infarction appeared to be the main contributors to the increased risk of MACE (Table 3).

**TABLE 3:** Joint association of TyG and hsCRP with endpoint events.

	Model 1				Model 2				Model 3		
	HR	95% CI	*p* value		HR	95% CI	*p* value		HR	95% CI	*p* value
MACE											
TyG T1 and hsCRP ≤3 mg/L	Ref	‐	‐		Ref	‐	‐		Ref	‐	‐
TyG T2 and hsCRP ≤3 mg/L	1.099	0.879–1.374	0.407		1.104	0.882–1.382	0.387		1.117	0.886–1.407	0.350
TyG T3 and hsCRP ≤3 mg/L	1.249	1.003–1.556	0.047*		1.227	0.978–1.539	0.077		1.180	0.927–1.504	0.179
TyG T1 and hsCRP >3 mg/L	1.162	0.820–1.646	0.399		1.139	0.804–1.614	0.464		1.004	0.697–1.445	0.985
TyG T2 and hsCRP >3 mg/L	1.339	0.986–1.820	0.062		1.329	0.977–1.808	0.070		1.220	0.887–1.679	0.222
TyG T3 and hsCRP >3 mg/L	1.819	1.398–2.366	<0.001*		1.803	1.376–2.362	<0.001*		1.577	1.180–2.108	0.002*
All‐cause death											
TyG T1 and hsCRP ≤3 mg/L	Ref	‐	‐		Ref	‐	‐		Ref	‐	‐
TyG T2 and hsCRP ≤3 mg/L	1.211	0.723–2.029	0.467		1.337	0.795–2.249	0.274		1.538	0.891–2.655	0.122
TyG T3 and hsCRP ≤3 mg/L	0.988	0.570–1.712	0.965		1.133	0.643–1.997	0.665		1.206	0.659–2.207	0.544
TyG T1 and hsCRP >3 mg/L	2.261	1.186–4.312	0.013*		2.085	1.093–3.978	0.026*		1.951	0.976–3.899	0.058
TyG T2 and hsCRP >3 mg/L	2.668	1.497–4.757	0.001*		2.708	1.514–4.845	0.001*		2.696	1.448–5.016	0.002*
TyG T3 and hsCRP >3 mg/L	2.841	1.649–4.894	<0.001*		3.220	1.831–5.665	<0.001*		2.732	1.466–5.094	0.002*
Cardiac death											
TyG T1 and hsCRP ≤3 mg/L	Ref	‐	‐		Ref	‐	‐		Ref	‐	‐
TyG T2 and hsCRP ≤3 mg/L	0.931	0.465–1.864	0.840		1.000	0.497–2.012	1.000		1.317	0.626–2.770	0.469
TyG T3 and hsCRP ≤3 mg/L	1.111	0.567–2.176	0.759		1.218	0.608–2.439	0.578		1.537	0.718–3.292	0.268
TyG T1 and hsCRP >3 mg/L	1.794	0.744–4.327	0.193		1.670	0.692–4.028	0.254		1.374	0.511–3.697	0.529
TyG T2 and hsCRP >3 mg/L	2.754	1.338–5.671	0.006*		2.747	1.328–5.679	0.006*		3.069	1.401–6.721	0.005*
TyG T3 and hsCRP >3 mg/L	2.526	1.245–5.124	0.010*		2.720	1.306–5.667	0.008*		2.765	1.231–6.212	0.014*
Nonfatal myocardial infarction											
TyG T1 and hsCRP ≤3 mg/L	Ref	‐	‐		Ref	‐	‐		Ref	‐	‐
TyG T2 and hsCRP ≤3 mg/L	0.914	0.435–1.921	0.812		0.912	0.433–1.921	0.808		1.140	0.528–2.462	0.738
TyG T3 and hsCRP ≤3 mg/L	1.336	0.673–2.651	0.407		1.291	0.638–2.612	0.477		1.681	0.791–3.575	0.177
TyG T1 and hsCRP >3 mg/L	2.339	0.992–5.517	0.052		2.295	0.973–5.418	0.058		2.051	0.817–5.145	0.126
TyG T2 and hsCRP >3 mg/L	2.411	1.083–5.366	0.031*		2.360	1.057–5.269	0.036*		2.568	1.112–5.930	0.027*
TyG T3 and hsCRP >3 mg/L	3.078	1.505–6.296	0.002*		2.972	1.420–6.220	0.004*		3.578	1.596–8.018	0.002*
Revascularization											
TyG T1 and hsCRP ≤3 mg/L	Ref	‐	‐		Ref	‐	‐		Ref	‐	‐
TyG T2 and hsCRP ≤3 mg/L	1.101	0.857–1.415	0.450		1.089	0.847–1.401	0.506		1.069	0.825–1.384	0.615
TyG T3 and hsCRP ≤3 mg/L	1.300	1.018–1.659	0.035		1.235	0.960–1.589	0.101		1.165	0.889–1.525	0.268
TyG T1 and hsCRP >3 mg/L	0.911	0.592–1.401	0.671		0.906	0.589–1.395	0.655		0.788	0.502–1.237	0.300
TyG T2 and hsCRP >3 mg/L	1.082	0.746–1.570	0.678		1.071	0.737–1.556	0.719		0.988	0.673–1.453	0.953
TyG T3 and hsCRP >3 mg/L	1.637	1.206–2.220	0.002*		1.573	1.151–2.151	0.005*		1.387	0.992–1.941	0.056

*Note*: Model 1: univariate model for TyG and hsCRP. Model 2: adjusted for sex, age, and DM. Model 3: adjusted for sex, age, BMI, hypertension, cerebrovascular disease, peripheral vascular diseases, anemia, DM, previous myocardial infarction, previous PCI, previous CABG, LDL‐C, creatine, albumin, HbA1c, LVEF, LM/TVD, and SYNTAX score.

Abbreviations: BMI, body mass index; CABG, coronary artery bypass grafting; CI, confidence interval; DM, diabetes mellitus; HbA1c, glycosylated hemoglobin A1c; HR, hazard ratio; hsCRP, high‐sensitivity C‐reactive protein; LDL‐C, low‐density lipoprotein cholesterol; LM/TVD, left main or three‐vessel disease; LVEF, left ventricular ejection fraction; MACE, major adverse cardiovascular events; PCI, percutaneous coronary intervention; SYNTAX, synergy between PCI with taxus and cardiac surgery; TyG, triglyceride–glucose.

* *p* value <0.05.

No significant multiplicative or additive interaction was found between TyG and hsCRP on the risk of MACE, with multiplicative effect = 0.977 (95% CI: 0.950–1.004, *p* = 0.099), RERI = 0.180 (95% CI: −0.100 to 0.368), AP = 0.126 (95% CI: −0.043 to 0.288), and SI = 1.744 (95% CI: 0.551–5.515).

### 3.4 Sensitivity analysis

**FIGURE 6:**
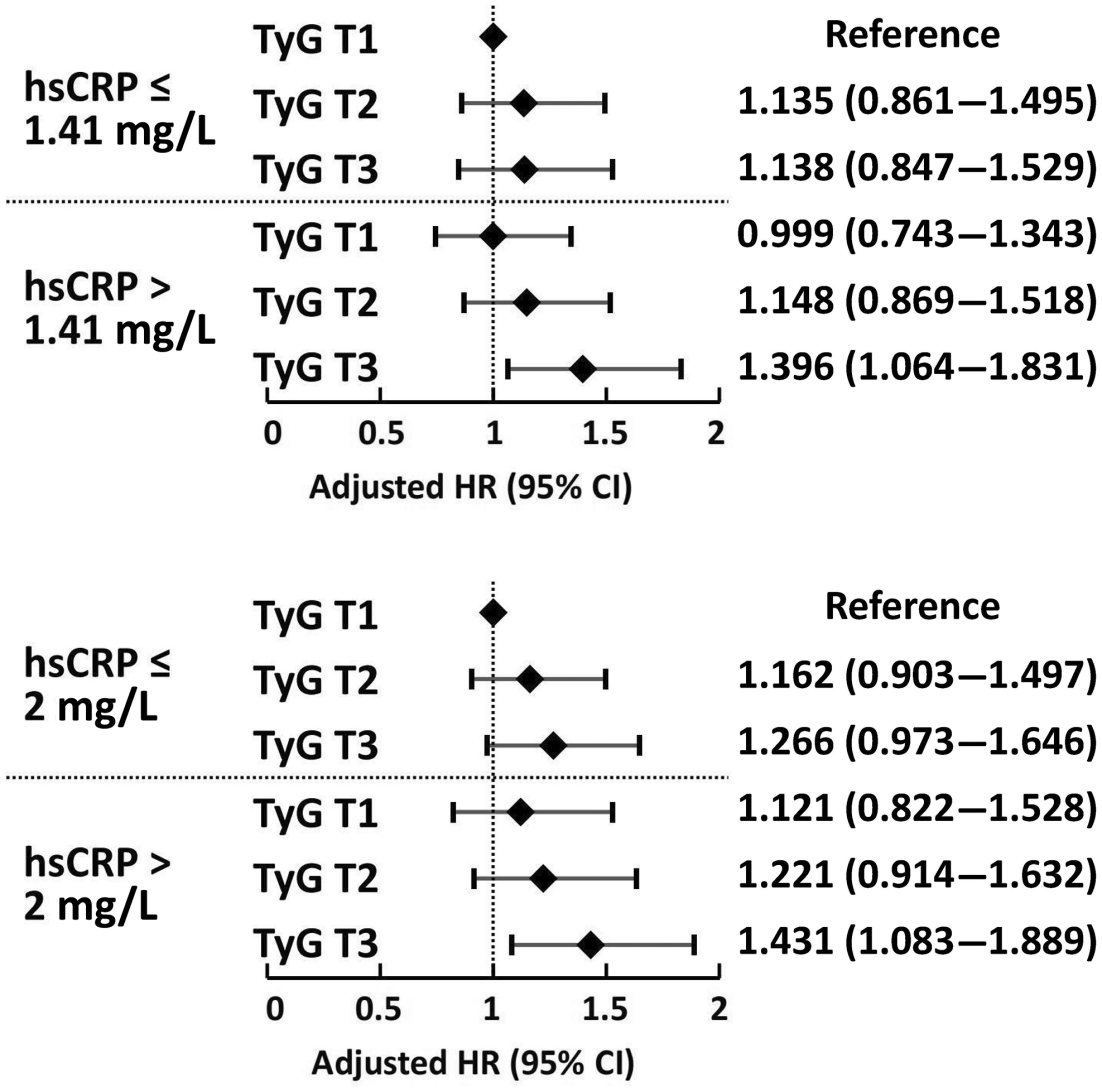
Sensitivity analysis of regrouping according to different high‐sensitivity C‐reactive protein (hsCRP) levels. CI, confidence interval; HR, hazard ratio; TyG, triglyceride–glucose.

Participants were regrouped according to the median level and 2 mg/L of hsCRP, and the results showed that the TyG T3 and hsCRP >1.41 mg/L group and the TyG T3 and hsCRP >2 mg/L group had a significantly higher risk of MACE (Figure 6), which was consistent with the previous results.

## 4 DISCUSSION

This study investigated the combined relationship between TyG and hsCRP with MACE during 2‐year follow‐up in patients with chronic coronary syndrome. We found that elevated TyG levels were associated with a higher risk of MACE in patients with CCS after adjusting for potential confounding risk factors. It is noteworthy that among patients in the highest tertile of TyG, hsCRP >3 mg/L was significantly associated with an increased risk of MACE, whereas the results were not significant in the medium to low TyG groups. In addition, participants with both elevated TyG and hsCRP levels suffered the highest risk of MACE. Our study suggests that simultaneous assessment of TyG and hsCRP may help identify high‐risk populations and guiding management strategies among patients with CCS.

Despite the gradual improvement in pharmacological and revascularization therapies for patients with CCS, they still remain at a higher risk of experiencing adverse cardiovascular events.^1^ The identification and targeted management of residual risks are crucial for improving patient prognosis. Insulin resistance stands as a significant risk factor for the development of CAD.^4^, ^25^ The TyG index, a simple surrogate marker of insulin resistance, has been shown to have a similar ability to evaluate insulin sensitivity as the hyperinsulinemic–euglycemic clamp.^26^ Prior investigations have demonstrated an association between the TyG index and MACE. Elevated TyG index has been observed to correlate with an augmented occurrence of CAD and adverse cardiac events within the general population without diagnosed cardiovascular diseases.^7^, ^8^, ^27^, ^28^, ^29^ In terms of the patients with confirmed cardiovascular diseases, current research has shown that the TyG index is capable of independently predicting the risk of adverse cardiovascular events in patients with ACS, regardless of their diabetic status.^10^, ^11^, ^30^, ^31^ CAD is a dynamic process characterized by the accumulation of atherosclerotic plaques and alterations in coronary circulation function. Myocardial infarction development may result in stress‐induced hyperglycemia, potentially influencing the predictive value of the TyG index. However, there is currently limited research on patients with CCS. A nested case‐control analysis study has revealed that patients with stable CAD (SCAD) who experience adverse cardiovascular events exhibit elevated TyG index levels, and the TyG index is an independent marker for assessing the risk of adverse cardiovascular events in patients with SCAD.^9^ Another retrospective observational study has demonstrated a significant association between elevated TyG index and an increased risk of MACE in patients with both combined CCS and coronary microvascular dysfunction.^32^ Our study observed consistent results, indicating that an elevated TyG index is independently associated with MACE in patients with CCS, further supporting the prognostic value of the TyG index in this specific patient population.

Systemic inflammation is a significant contributor to the occurrence and progression of CAD. HsCRP has become a commonly used biomarker for inflammation risk in CAD because of its clinical availability and association with the risk of adverse cardiovascular events.^33^ Several prospective cohort studies and clinical trials have demonstrated the association of hsCRP with adverse outcomes in patients with stable atherosclerotic cardiovascular disease.^13^, ^34^, ^35^ Our study obtained the same results. A recent analysis of three randomized clinical trials revealed that hsCRP outperformed LDL‐C in predicting future cardiovascular events and mortality in patients receiving statin therapy.^36^ The Canankinumab Anti‐inflammatory Thrombosis Outcomes Trial demonstrated a significant reduction in hsCRP levels with the interleukin‐1β‐targeted drug canakinumab.^37^ Furthermore, a concentration of hsCRP below 2 mg/L at 3 months after canakinumab treatment was associated with a significant decrease in the occurrence of adverse cardiovascular events, and the greatest reduction in the adverse cardiovascular event risk was observed in patients with the largest decrease in hsCRP levels.^38^ It is crucial to promptly assess the residual inflammation risk in patients with CAD and explore anti‐inflammatory therapies to further mitigate cardiovascular risk. However, the selection criteria for anti‐inflammatory therapy in CCS patients remain unclear. The results of our study indicated that among patients in the highest TyG group, those with hsCRP >3 mg/L had a significantly higher risk of MACE compared with those with hsCRP ≤3 mg/L. In contrast, no significant difference in MACE risk was observed between the two groups of patients stratified by hsCRP in the mid–low TyG group. Therefore, among patients with CCS, those with elevated levels of both hsCRP and TyG may potentially benefit from anti‐inflammatory therapy to improve prognosis.

The mechanism of the association between inflammation and insulin resistance has not been fully elucidated. The available data support the idea that there is a reciprocal relationship between chronic inflammation and insulin resistance. Inflammation may promote insulin resistance through proinflammatory cytokines, such as interleukin‐1β and tumor necrosis factor‐α, as well as adipose tissue‐specific macrophages.^17^, ^18^, ^39^ On the other hand, insulin resistance may induce inflammation through increased free fatty acid oxidation and reactive oxygen species formation.^40^ It has been shown that elevated hsCRP is associated with insulin resistance in nondiabetic adults.^41^, ^42^ However, the existing evidence concerning the association between TyG index, hsCRP, and cardiovascular risk is currently limited. A cross‐sectional study demonstrated that the combination of TyG and hsCRP may better identify moderate‐to‐severe asymptomatic intracranial arterial stenosis.^43^ Another study conducted on middle‐aged and elderly Chinese individuals without a baseline history of cardiovascular disease revealed that the participants who exhibited concurrent elevations in hsCRP and TyG had a higher risk of developing cardiovascular disease (CVD) compared with those with lower levels of both hsCRP and TyG. The incorporation of both hsCRP and TyG into traditional risk models resulted in a significant enhancement of risk stratification for CVD. However, they found no interaction between hsCRP and the TyG on CVD.^19^ Our study further investigated the multiplicative and additive interactions between TyG and hsCRP on MACE risk. However, similarly, no significant findings were observed. This observation adds complexity to understanding how TyG and hsCRP jointly influence MACE risk in CCS patients. Further research is needed to elucidate the mechanisms by which TyG and hsCRP contribute to adverse cardiovascular events. Nonetheless, our study does underscore the importance of jointly assessing hsCRP and TyG in CCS patients. Our study showed that individuals with simultaneous elevations in hsCRP and TyG index experienced the highest risk of MACE. Consequently, the simultaneous evaluation of TyG index and hsCRP may facilitate improved risk stratification and the identification of high‐risk individuals among CCS patients, thereby guiding the implementation of precise and tailored treatment strategies.

This study has several limitations. First, some patients were excluded because of missing data or loss to follow‐up, potentially affecting the results. Although we performed statistical adjustments for most confounding factors in the multivariable regression, there could still be unaccounted variables, and the adjustments may not completely eliminate the confounding. Second, the prehospitalization medication status of patients might influence the levels of TyG and hsCRP, but this aspect was not addressed because of insufficient data. The TyG index and hsCRP were measured only once at baseline, and the use of lipid‐lowering and glucose‐lowering medications during follow‐up could impact these biomarkers. Further dynamic monitoring of TyG index and hsCRP levels is necessary to assess their average levels, variability, and their association with adverse cardiovascular outcomes. Lastly, this study focused on hospitalized patients with coronary heart disease who were not routinely tested for insulin levels, and therefore, a comparison between the homeostatic model assessment for insulin resistance and the TyG index was not conducted.

## 5 CONCLUSIONS

Participants with concurrently elevated hsCRP and TyG had worse clinical outcomes. The concurrent assessment of TyG and hsCRP may be valuable in identifying high‐risk groups and guiding management strategies among patients with CCS.

## AUTHOR CONTRIBUTIONS

J.Y., Y.H., and Q.L. contributed to the conception and design of the work. Y.S., Z.Z., J.X., Z.L., X.T., X.W., Y.C., Y.Z., P.Z., X.G., L.J., Z.W., R.L., Q.W., Y.Y., and Y.F. contributed to data collection and analysis. Q.L. drafted the manuscript. J.Y. and Y.H. critically revised the manuscript. All authors read and approved the final manuscript.

## FUNDING INFORMATION

This research was supported by the China National Key R&D Program during the 13th Five‐Year Plan Period, grant numbers 2016YFC1301300, 2016YFC1301301; the Chinese Academy of Medical Sciences (CAMS) Innovation Fund for Medical Sciences (CIFMS), grant number 2023‐I2M‐1‐002; and National Clinical Research Center for Cardiovascular Diseases, Fuwai Hospital, Chinese Academy of Medical Sciences, grant number NCRC2020013.

## CONFLICT OF INTEREST STATEMENT

The authors declare no conflicts of interest.
